# Obsessive–compulsive symptoms are negatively correlated with motor severity in patients with generalized dystonia

**DOI:** 10.1038/s41598-022-24826-x

**Published:** 2022-11-27

**Authors:** Taku Matsuda, Ryoma Morigaki, Yuki Matsumoto, Hideo Mure, Kazuhisa Miyake, Masahito Nakataki, Masafumi Harada, Yasushi Takagi

**Affiliations:** 1 Department of Neurosurgery, Graduate School of Biomedical Sciences, Tokushima University, 3-18-15, Kuramoto-cho, Tokushima, 770-8503 Japan; 2 Department of Advanced Brain Research, Graduate School of Biomedical Sciences, Tokushima University, Tokushima, 770-8503 Japan; 3 Department of Radiology and Radiation Oncology, Graduate School of Biomedical Sciences, Tokushima University, Tokushima, 770-8503 Japan; 4 Center for Neuromodulation, Department of Neurosurgery, Kurashiki Heisei Hospital, Okayama, 710-0826 Japan; 5 Department of Psychiatry, Graduate School of Biomedical Sciences, Tokushima University, Tokushima, 770-8503 Japan

**Keywords:** Dystonia, Movement disorders, Psychiatric disorders

## Abstract

We aimed to clarify the correlations between motor symptoms and obsessive–compulsive symptoms and between the volumes of basal ganglia components and obsessive–compulsive symptoms. We retrospectively included 14 patients with medically intractable, moderate and severe generalized dystonia. The Burke–Fahn–Marsden Dystonia Rating Scale and Maudsley Obsessional Compulsive Inventory were used to evaluate the severity of dystonia and obsessive–compulsive symptoms, respectively. Patients with generalized dystonia were divided into two groups; patients whose Maudsley Obsessional Compulsive Inventory score was lower than 13 (Group 1) and 13 or more (Group 2). Additionally, the total Maudsley Obsessional Compulsive Inventory scores in patients with dystonia were significantly higher than normal volunteers’ scores (*p* = 0.025). Unexpectedly, Group 2 (high Maudsley Obsessional Compulsive Inventory scores) showed milder motor symptoms than Group 1 (low Maudsley Obsessional Compulsive Inventory scores) (*p* = 0.016). “Checking” rituals had a strong and significant negative correlation with the Burke–Fahn–Marsden Dystonia Rating Scale (ρ = − 0.71, *p* = 0.024) and a strong positive correlation with the volumes of both sides of the nucleus accumbens (right: ρ = 0.72, *p* = 0.023; left: ρ = 0.70, *p* = 0.034). Our results may provide insights into the pathogenesis of obsessive–compulsive disorder and dystonia.

## Introduction

Obsessive–compulsive disorder (OCD) is characterized by repetitive, intrusive, and persistent thoughts and behaviors^1^. Some of the clinical characteristics of OCD include checking compulsions, symmetry obsessions, contamination obsessions, repugnant obsessions, and hoarding obsessions^2,3^. There are a few types of test batteries that have been used for the evaluation of these symptoms, such as the Maudsley Obsessional Compulsive Inventory (MOCI), which is a 30-item self-report questionnaire that can easily assess OCD severity^4^. The items are divided into four subclasses: “Checking,” “Cleaning,” “Slowness,” and “Doubting”^5^. The first-line treatments for OCD are pharmacotherapy and cognitive-behavioral therapy, but 10% of patients are refractory to these treatments^6^. The pathophysiology of OCD is thought to be a dysfunction of the cortical-striato-thalamo-cortical loop, which is also termed the OCD loop^7^. Deep brain stimulation (DBS) is performed in the United States of America and some European countries to treat patients with refractory OCD by modulating the OCD loop^8^. The structures selected and targeted by DBS for the treatment of OCD are the anterior limb of the internal capsule, ventral capsule, ventral striatum, nucleus accumbens (NAc), and subthalamic nucleus^9^. Some reports indicate that different loops are associated with different OCD symptoms^10,11^. There are three commonly defined OCD loops: the “affective circuit,” which consists of the anterior cingulate cortex (ACC)/ventromedial prefrontal cortex and NAc; the “ventral cognitive circuit,” which consists of the anterolateral orbitofrontal cortex and putamen; and the “dorsal cognitive circuit,” which consists of the dorsolateral prefrontal cortex and caudate^12^.

Dystonia is a movement disorder characterized by sustained muscle contractions that generate twisting, repetitive or patterned movement, and abnormal postures^13^. The Burke–Fahn–Marsden Dystonia Rating Scale (BFMDRS) is one of the most reported measures for assessing the severity of dystonia^14,15^. The main cause of dystonia is also proposed to be dysfunction of the cortico-basal ganglia-thalamo-cortical loop^16^. The effectiveness of pallidal-DBS for medically refractory dystonia supports this notion^17^.

The main difference in the cortico-basal ganglia-thalamo-cortical loop between OCD and dystonia, though their loops are similar to each other, could be the functional subdivisions in the same structures involved in each loop. For example, the dorsolateral striatum (motor part) is predominantly related to dystonic symptoms, whereas the ventral striatum (limbic part) is predominantly related to OCD^18,19^.

The relationship between movement disorders and psychiatric disorders is still under debate^20^. Graybiel suggested that rituals and actions might have common neural mechanisms in movement disorders that accompany stereotypies, such as Huntington’s disease, dystonia, and Parkinson’s disease^21^. Some reports suggest a correlation between dystonia and OCD. The incidence of OCD is greater in patients with myoclonus-dystonia^22,23^ or focal dystonia^24–26^; however, OCD shows no association with DYT1 mutation carriers or patients manifesting DYT1 dystonia^27^. We hypothesize that in patients with dystonia, there will be a correlation between the severity of dystonia and the presence of obsessive–compulsive symptoms as measured by their BFMDRS and MOCI scores.

## Results

During the inclusion period, 23 patients underwent globus pallidus internus DBS for generalized dystonia. Nine patients were excluded, as 4 patients were not able to communicate with others because of severe dystonia, and data were missing for the other 5 patients. The characteristics of the 14 remaining patients included in the study and comparisons to normal volunteers are shown in Table 1. The median age of these patients was 35 years, the median disease duration was 13.0 years, the median MOCI score was 8.5 (5.0–14.3), and the median BFMDRS score was 54.5 (43.8–64.5). Four patients had an MOCI score of ≥ 13. The total MOCI scores in generalized dystonia patients were higher than those in normal volunteers (*p* = 0.025) (Table 1). Pre-and post-operative MOCI scores were only available for 10 patients as 4 patients were followed up in other institutions; there were no significant changes between pre-and post-operative MOCI scores (*p* = 0.953) for these 10 patients.

**Table 1:** Comparisons between the characteristics of patients with dystonia and normal volunteers.

	Dystonia patients	Normal volunteers	*p* value
Age, years [median (interquartile range)]	35.0 (24.3–50.0)	35.0 (22.3–52.0)	0.890
Sex, F (%)/M (%)	8 (57)/6 (43)	8 (57)/6 (43)	1.000
Disease duration, years [median (interquartile range)]	13.0 (7.8–20.0)	–	–
Total MOCI score [median (interquartile range)]	8.5 (5.0–14.3)	4.0 (2.0–7.5)	0.025*
Total MOCI score ≥ 13 (%)	4 (29)	0 (0)	0.098
BFMDRS [median (interquartile range)]	54.5 (43.8–64.5)	–	–

A *p* value < 0.05 is statistically significant.

BFMDRS, Burke–Fahn–Marsden Dystonia Rating Scale; F, female; M, male; MOCI, Maudsley Obsessional Compulsive Inventory.

There were no significant differences in age, sex, and disease duration between generalized dystonia patients whose total MOCI score was lower than 13 (Group 1) and 13 or more (Group 2). The BFMDRS was lower in Group 2 (high MOCI) (median, 28.5; interquartile range, 19.3–46.0) than in Group 1 (low MOCI) (median, 58.8; interquartile range, 50.4–68.0) (*p* = 0.016) (Supplementary Table 1), indicating that severe obsessive symptoms are associated with milder dystonia.

**Figure 1:**
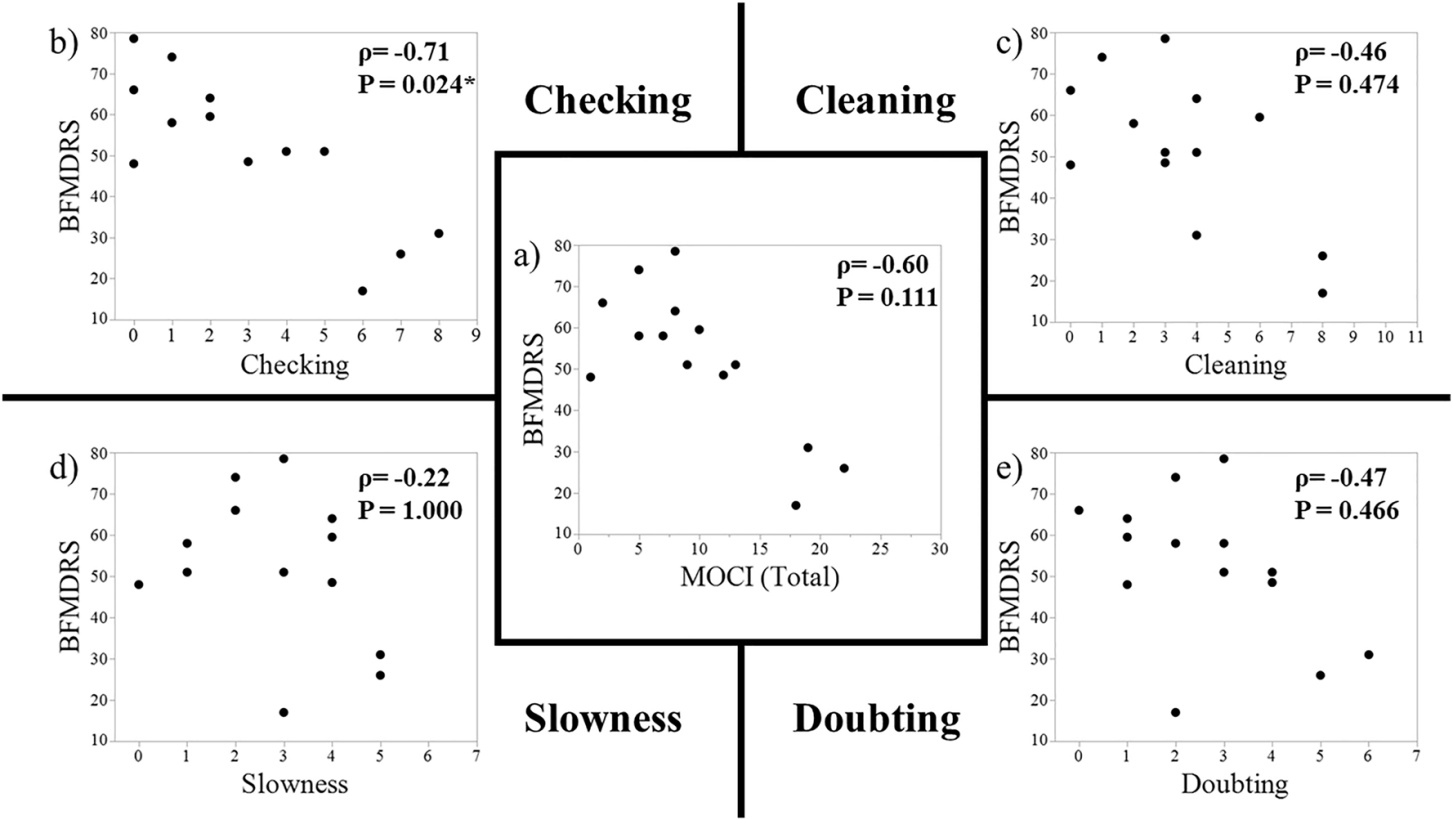
Correlations between the BFMDRS and the total MOCI score (**a**) and the scores of the four MOCI subscales (**b**–**e**). The correlation coefficient (ρ) and corresponding *p* values (*p*) are shown. A *p* value < 0.05 is statistically significant. BFMDRS, Burke–Fahn–Marsden Dystonia Rating Scale; MOCI, Maudsley Obsessional Compulsive Inventory.

Next, we examined the correlation between the BFMDRS and the total MOCI score and the scores of its four subscales. The MOCI scores did not have any significant relationship with the BFMDRS except for the “Checking” score, which had a strong significant negative correlation (ρ = − 0.71, *p* = 0.024) (Fig. 1); the more checking rituals the patients had, the milder their dystonic symptoms were.

**Figure 2:**
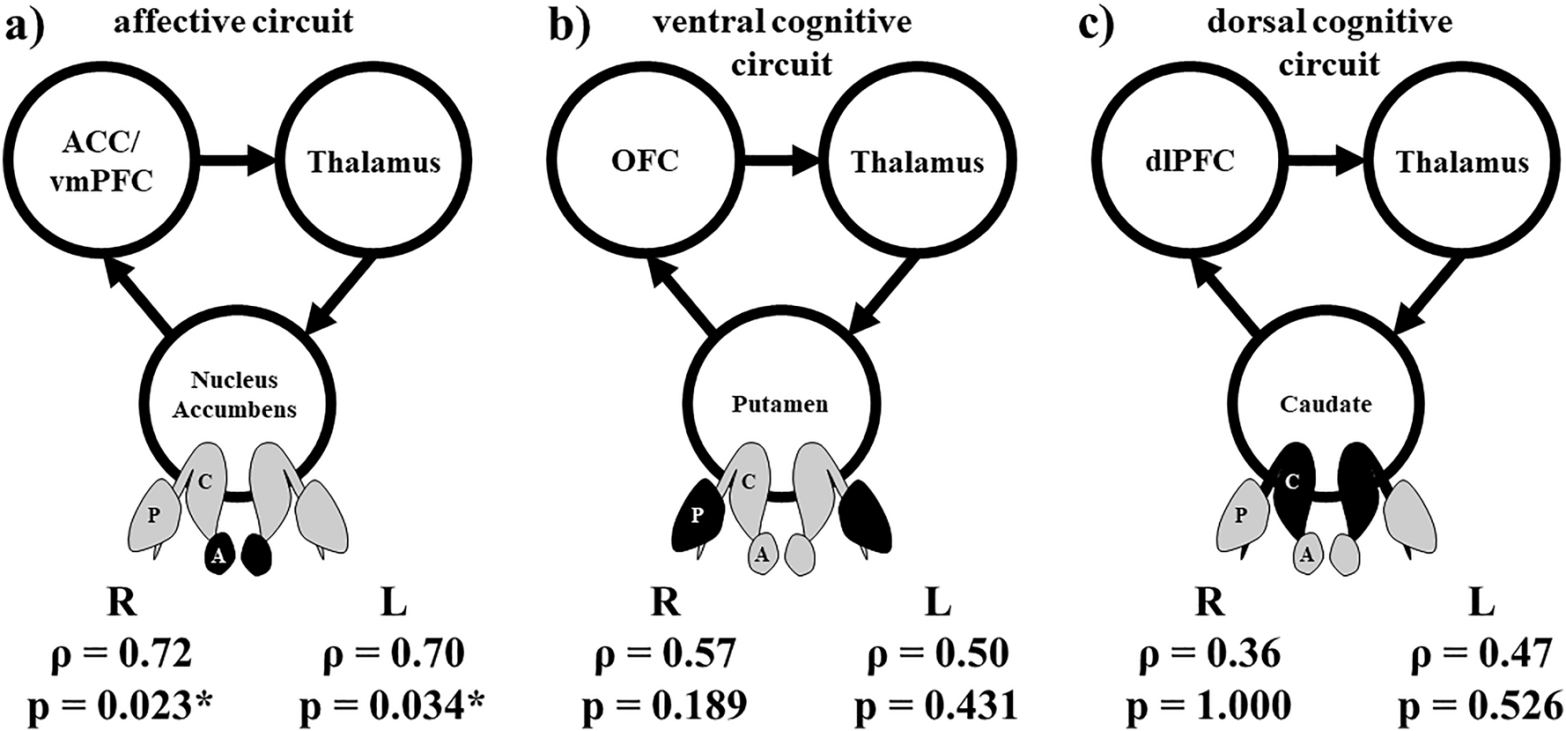
The correlation (ρ) between the “Checking” scores and the volumes of the striatal components of the three major OCD loops^12^. (**a**) Nucleus accumbens, (**b**) putamen, and (**c**) caudate. A *p* value < 0.05 is statistically significant. ACC, anterior cingulate cortex; dlPFC, dorsolateral prefrontal cortex; L, left; OFC, orbitofrontal cortex; R, right; vmPFC, ventromedial prefrontal cortex.

We used the NAc, putamen, and caudate nucleus as representative structures of the three OCD loops mentioned previously. These structures were chosen because a previous report determined that these structures are the main “striato” parts of the OCD loop^12^. Because the “Checking” score had a strong negative correlation with the BFMDRS, we investigated whether this subscale was associated with the volumes of these structures. We found a significant strong positive correlation between the “Checking” score and the volume of each side of the NAc (right: ρ = 0.72, *p* = 0.023; left: ρ = 0.70, *p* = 0.034). On the contrary, there were no significant correlations between the “Checking” score and the volumes of either side of the putamen or caudate (Fig. 2, Supplementary Figure 1).

## Discussion

We reported that the severity of OCD in patients with generalized dystonia was higher than that in normal volunteers and subsequently showed that the “Checking” items of obsessive–compulsive symptoms were negatively correlated with the severity of dystonia in patients with generalized dystonia. Intriguingly, the “Checking” score of obsessive–compulsive ideas was positively correlated with the volumes of both sides of the NAc.

We chose patients with generalized dystonia because the entire motor loop was assumed to be involved, consequently, the patients had moderate to severe dystonia. Some studies have reported that the rate of OCD is high among patients with myoclonus-dystonia^22,23^ or focal dystonia^24–26^. These patients have segmental or focal dystonia, and the whole motor loop might not be involved. One study has reported that OCD is not associated with DYT1 generalized dystonia^27^. Our report is the first to show a correlation between generalized dystonia and obsessive–compulsive symptoms.

We showed an unexpected negative correlation between the score of the “Checking” ritual and the severity score of dystonia. The other three subscales, “Cleaning,” “Slowness,” and “Doubting” rituals, did not have any significant correlations with dystonia severity. Furthermore, we confirmed that the score of “Checking” rituals had a positive correlation with the volumes of both sides of the NAc. Piras et al. assumed that the increase in the volume of deep gray matter in patients with OCD might reflect hyperactivity in the OCD loop^28^. Likewise, Bai et al. hypothesized that the increase in the volume of the globus pallidus in patients with primary dystonia might suggest globus pallidus over-activation^29^. Murayama et al. have reported that the ACC is related more to the severity of “Checking” rituals than to that of “Washing” rituals^10^. Jung et al. reported that there was a significant positive correlation between ventral striatal activation and compulsion symptom severity^11^. We assumed that the repetitive “Checking” ritual was associated with hyperactivity of the ACC-NAc-thalamo-ACC loop and might increase the volume of the NAc as a result of the neuroplastic change. One study reported that the volume of the right NAc has a negative relationship with the severity of OCD^30^, but our results contradict this. Although the precise reason is unknown, the discrepancy might, in part, be due to methodological differences. In their report, patients diagnosed with OCD were included. In contrast, we only enrolled patients with generalized dystonia.

Several reports have suggested that parvalbumin interneurons in the striatum are suppressed in animal models of dystonia^31–33^ and OCD^34,35^. Dysfunction of parvalbumin interneurons in the striatum may cause dystonia and OCD. Sciamanna et al. reported that corticostriatal and thalamostriatal inputs activate or inhibit parvalbumin interneurons in the striatum, respectively^36^. Although the precise reason is unknown, the degree of suppression of parvalbumin interneurons might be different between the dorsolateral motor striatum and the limbic nucleus accumbens, which might explain why our results indicate that the severity of dystonia is negatively correlated with the “Checking” ritual in patients with generalized dystonia.

There are some limitations to our study. We examined only severe generalized dystonia; therefore, the relationship between mild dystonia and other types of dystonia and obsessive–compulsive symptoms remains unclear. Second, the number of patients was small. Generalized dystonia is a rare disease, and thus, it was difficult to enroll an adequate number of patients. Third, the number of patients whose MOCI score was 13 or higher (Group2) was small because this study focused on generalized dystonia, not OCD. Fourth, OCD was not diagnosed correctly because we used MOCI instead of the Diagnostic and Statistical Manual of Mental Disorders (DSM) or International Classification of Disease. The only report that MOCI might be valid for screening OCD was verified by DSM-III-R, which had been superseded by DSM-V^37^. Therefore, we did not use MOCI for diagnosing purposes and applied it to the severity cut-off point. However, the cut-off point might be different if we use DSM-V.

In conclusion, our study findings indicate a negative correlation between the severity of generalized dystonia and “Checking” rituals in obsessive–compulsive symptoms. “Checking” rituals were positively correlated with the volume of each side of the NAc. This result supports that there is a relationship between generalized dystonia and obsessive–compulsive symptoms. Longitudinal, prospective, and multicenter studies are needed; however, our findings provide key evidence for understanding the relationship between psychiatric and movement disorders.

## Methods

We retrospectively included patients with medically intractable moderate and severe generalized dystonia who underwent DBS between April 2012 and December 2019 at Tokushima University Hospital. The classification of dystonia was determined according to a report by Albanese et al.^13^. Patients who were unable to communicate with others or who had missing pre-operative data were excluded. We used the BFMDRS and the Japanese version of the MOCI to evaluate the severity of dystonia and obsessive–compulsive symptoms, respectively. The patients answered MOCI battery by themselves and they were blind to the type of the battery when they performed the questionnaires. Two neurosurgeons, who have treated movement disorders for more than 10 years and were blinded to the MOCI score, evaluated pre-and post-operative motor symptoms using the BFMDRS. Post-operative scores were examined more than 1 month after surgery (Supplementary Table 2). If there was a discrepancy in scoring, the assessors had a discussion to reach a consensus and decided on the final score. Because the MOCI scores of healthy people are unknown, the MOCI scores of 14 normal volunteers who were matched for sex and age to patients were also determined (Supplementary Table 3). Written informed consent was obtained from all participants according to the Declaration of Helsinki, and the study was approved by the ethical committee of Tokushima University Hospital (number: 3743–1). The four subscales of the MOCI, “Checking” (9 items), “Cleaning” (11 items), “Slowness” (7 items), and “Doubting” (7 items)^5^ were investigated. Tadai et al. reported that when the cut-off point of the total MOCI score was set to 12/13, OCD could be diagnosed with high sensitivity (100%) and specificity (96.0%)^37^; therefore, we adopted this cut-off point. Since this cut-off point was determined by DSM-III-R, we cannot simply apply it to the current diagnosis criteria (DSM-V) of OCD. However, this cut-off point has been adopted by other reports^38,39^. The patients were divided into two groups: patients whose total MOCI score was lower than 13 (Group 1) and 13 or higher (Group 2).

Magnetic resonance imaging was performed before surgery to determine the coordinates of the target; T1-weighted gradient echo imaging (GE Healthcare, USA) was used for this purpose and its slice thickness was set at 0.7–0.9 mm. The volumes of each side of the NAc, putamen, and caudate were calculated using FreeSurfer software (version 7.1.1, http://surfer.nmr.mgh.harvard.edu). The segmentations and volumetric measurements of these structures were performed according to a previous report^40^. An example of segmentation is shown in Supplementary Figure 2. We examined the correlation of the BFMDRS with the volumes of each side of the brain segments and with the scores of the MOCI subscales.

### Statistical analysis

All statistical analyses were performed using JMP 14.0.0 (SAS Institute Inc., USA). When we compared the two groups, the Mann–Whitney U test or Wilcoxon signed-rank test was used for continuous variables, and Fisher’s exact test was used for categorical variables. Correlations between two variables were analyzed using Spearman’s rank correlation coefficients. Statistical significance was set at *p* < 0.05. Dunnett’s tests were used when multiple comparisons were performed, showing adjusted *p*-values.

## Supplementary Information

Supplementary Information.

## Data Availability

All data supporting the findings of this study are available in the article and its supplementary materials.
