# Supplementary material for: Obsessive–compulsive symptoms are negatively correlated with motor severity in patients with generalized dystonia
Source: Sci Rep. 2022 Nov 27;12:20350. doi: 10.1038/s41598-022-24826-x (PMC9701695; doi:10.1038/s41598-022-24826-x)
Supplement: Supplementary file 1 — Supplementary Information. [file 41598_2022_24826_MOESM1_ESM.pdf]

Supplementary Figure 1

The correlations between the “Checking” rituals and the volumes of each side of the nucleus accumbens (a, b), putamen (c, d), and caudate nucleus (e, f)

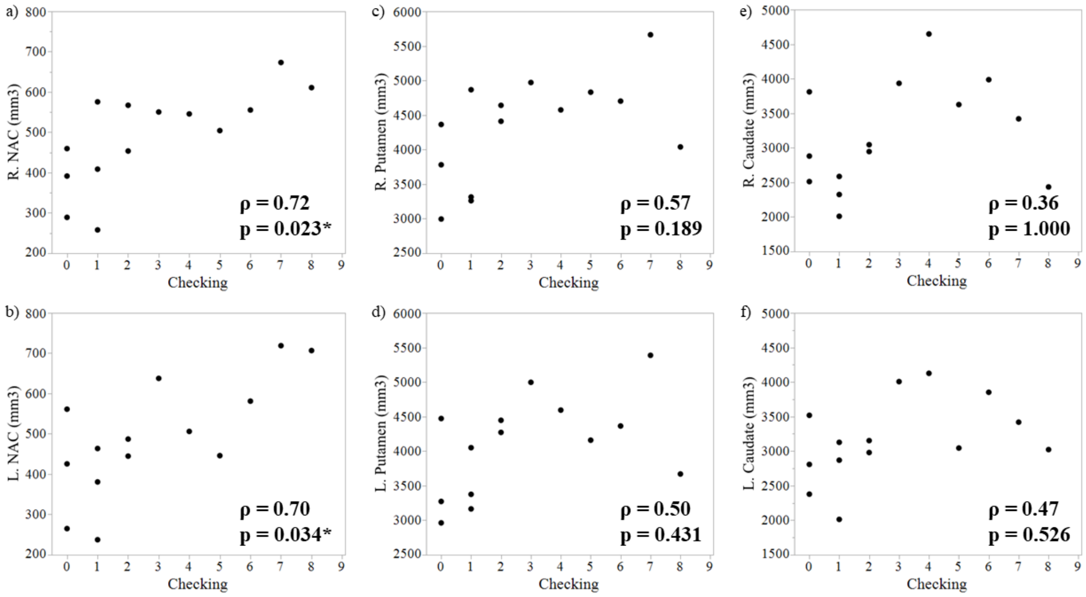

L, left; NAc, nucleus accumbens; R, right

Supplementary Figure 2

Example of the segmentation of the nucleus accumbens (A), putamen (P), and caudate (C)

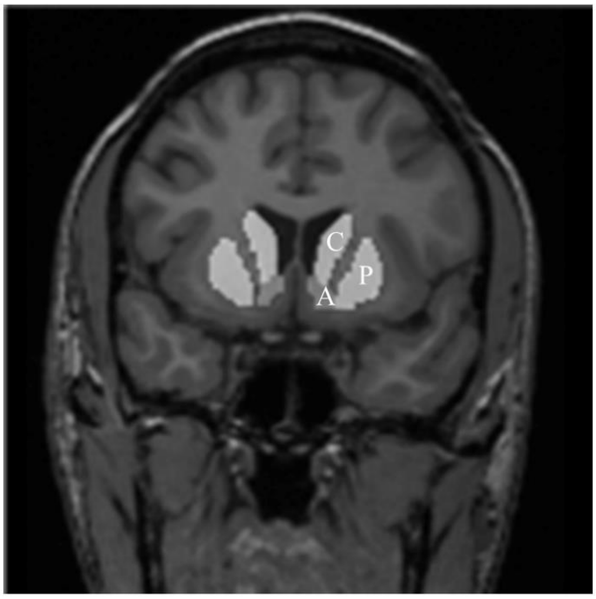

Supplementary Table 1: Comparisons between patients with generalized dystonia whose total MOCI score was lower than 13 (Group 1; low MOCI) and 13 or higher (Group 2; high MOCI)

	Group 1; low MOCI (n=10)	Group 2; high MOCI (n=4)	p value
Age, years [median (interquartile range)]	35.0 (28.5–50.0)	36.5 (22.8–55.5)	1.000
Sex, F (%)/M (%)	7 (70) / 3 (30)	1(25) /3 (75)	0.245
Disease duration, years [median (interquartile range)]	15.0 (9.5–20)	9.5 (2.5–24.8)	0.479
BFMDRS [median (interquartile range)]	58.8 (50.4–68.0)	28.5 (19.3–46.0)	0.016*

A p-value <0.05 is statistically significant.

BFMDRS, Burke–Fahn–Marsden Dystonia Rating Scale; F, Female; M, Male; MOCI, Maudsley Obsessional Compulsive Inventory

Supplementary Table 2: The clinical characteristics of the 14 patients with generalized dystonia who were included in this study.

Patient	Sex	Age	Disease Durations (years)	BFMDRS	MOCI						Volume (mm^3)					
					Total (pre)	Checking	Cleaning	Slowness	Doubting	Total (post)	NAC		Putamen		Caudate	
											R	L	R	L	R	L
1	M	34	28	58	7	1	2	1	3	-	258.1	237.5	3315.2	3164.9	2010.1	2013.0
2	F	12	8	66	2	0	0	2	0	-	459.8	425.7	3781.4	3272.8	2881.4	2808.6
3	M	34	19	58	5	1	2	1	2	7	408.8	380.9	4867.2	4050.7	2325.2	3128.4
4	M	25	7	26	22	7	8	5	5	17	673.4	718.9	5663.2	5387.4	3420.5	3419.7
5	F	56	10	74	5	1	1	2	2	4	575.8	463.8	3261.2	3376.0	2586.8	2870.0
6	F	69	18	51	9	4	3	1	3	-	545.8	506.2	4575.5	4594.3	4648.4	4127.2
7	F	36	5	59.5	10	2	6	4	1	10	453.8	444.9	4641.4	4447.1	3045.9	3153.1
8	F	32	23	78.5	8	0	3	3	3	9	289.2	265.1	2996.3	2962.7	2512.5	2378.4
9	M	58	29	17	18	6	8	3	2	10	555.5	581.3	4702.5	4364.5	3986.9	3852.5
10	F	18	16	64	8	2	4	4	1	11	567.3	487.2	4410.4	4272.3	2946.6	2980.5
11	M	48	12	31	19	8	4	5	6	20	611.0	706.8	4039.5	3670.2	2434.9	3023.0
12	M	39	12	48.5	12	3	3	4	4	12	550.6	637.8	4971.7	4996.7	3934.6	4007.0
13	F	22	1	51	13	5	4	3	4	13	504.4	446.1	4831.5	4159.4	3626.5	3045.6
14	F	48	14	48	1	0	0	0	1	-	391.7	561.4	4364.6	4472.7	3811.2	3519.1

BFMDRS, Burke-Fahn-Marsden Dystonia Rating Scale; R, right; L, left; MOCI, Maudsley Obsessional Compulsive Inventory; NAC, Nucleus Accumbens

Supplementary Table 3: The clinical characteristics of the 14 normal volunteers.

No.	Sex	Age	MOCI				
			Total	Checking	Cleaning	Slowness	Doubting
1	M	23	10	5	1	1	3
2	M	32	1	0	1	1	0
3	M	35	1	1	0	0	0
4	M	36	7	3	0	1	3
5	M	52	9	4	2	2	3
6	M	52	2	0	1	0	1
7	F	66	7	3	1	2	2
8	F	15	2	0	1	0	1
9	F	18	9	3	2	5	2
10	F	20	2	0	0	0	2
11	F	33	6	0	5	1	1
12	F	35	5	0	2	2	2
13	F	45	3	0	3	0	0
14	F	57	3	0	1	0	2

MOCI, Maudsley Obsessional Compulsive Inventory
